# Role of Autologous Micro-Fragmented Adipose Tissue in Osteoarthritis Treatment

**DOI:** 10.3390/jpm14060604

**Published:** 2024-06-06

**Authors:** Paolo Trentani, Elena Meredi, Paola Zarantonello, Alessandro Gennai

**Affiliations:** 1 Medipro Center Bologna, 40100 Bologna, Italy; trentani71@gmail.com; 2 Inail Insurance Medicine Center, 40100 Bologna, Italy; e.maredi@inail.it; 3 IRCCS Rizzoli Ortopaedic Institute Bologna-Argenta, 40100 Bologna, Italy; 4 Studio Gennai, 40100 Bologna, Italy

**Keywords:** osteoarthritis (OA), autologous micro-fragmented adipose tissue, intra-articular injection, stromal vascular fraction (SVF), pain reduction, regenerative medicine

## Abstract

Osteoarthritis (OA) is the most common complex musculoskeletal disorder, resulting from the degeneration of the articular cartilage and characterized by joint pain and dysfunction that culminate in progressive articular cartilage loss. We present our experience in the management of hip and knee OA by means of the intra-articular injection of fat micrograft, describing our approach, which was developed from the belief in the powerful reparative effect of autologous fat graft on damaged tissue, as well as its natural lubricating effect on the joints. Inclusion criteria were as follows: men and women, aged 20 to 80 years, that referred articular pain of the hips and/or knees, showing initial-stage degenerative OA. From October 2018 to July 2023, a total of 250 patients underwent treatment with the Sefficare^®^ device (SEFFILINE srl, Bologna, Italy). The Superficial Enhanced Fluid Fat Injection device was used to perform autologous regenerative treatments in a safe, standardized, easy, and effective way on 160 women, 64%, and 90 men, 36%. A total of 190 procedures (76%) involved the knees, with 20 patients who were bilaterally treated, while 60 procedures, all unilateral, involved the hips (24%). The mean age at treatment was 52.4 years. Before treatment, each patient had undergone X-rays and Magnetic Resonance Imaging (MRI) of the painful hip/knee to evaluate and grade the articular OA. Postoperatively, each patient was assessed after one, three, six, and twelve months. The donor site postoperative course was uneventful other than minimal discomfort. Clinically, the ROM (range of motion) of the treated knee/hip increased an average of 10 degrees 3 months after treatment, but the stiffness was reduced, as reported by the patients. The VAS (Visual Analog Scale) was submitted at 3, 6, and 12 months, demonstrating a progressive reduction of pain, with the best score obtained at six months postoperatively. In total, 85% of patients were satisfied one year after treatment, with a considerable improvement in pain and quality of life. The satisfactory outcome of this minimally invasive procedure indicates that the intra-articular injection of fat micrograft can replace or considerably delay the need for the classical major joint replacement surgery, thanks to its impact on the quality of life of patients and financial cost.

## 1. Introduction

Osteoarthritis (OA) is the most common complex musculoskeletal disorder, resulting from the degeneration of the articular cartilage and characterized by joint pain and dysfunction that culminate in progressive articular cartilage loss [1]. OA mainly involves the weight-bearing joints (i.e., knees and hips) due to chronic high stress. Risk factors for OA include age, heredity, lifestyle factors, obesity, and local conditions (such as biomechanical consequences of joint injury, joint laxity, or malalignment). Its occurrence is expected to increase exponentially as the world population ages and obesity increases [2]. The usual patient presentation is joint pain, swelling, morning joint stiffness, progressively restricted movements, and major disability, generally with a deterioration in quality of life [3]. Goals for conservatively treating OA are largely palliative and include approaches to relieve pain, to reduce the decline progression, to improve joint biomechanics, to increase muscle strength and conditioning, to delay total arthroplasty, and to preserve functional independence, mobility, and quality of life. Current treatments include physical therapy, weight loss, lifestyle changes, pharmacologic therapies, steroid injections, intra-articular hyaluronic acid injections, and, finally, surgery [4]. None of these treatments reverses or repairs the degenerative nature of the disease [5]. With this focus, stem cell therapies are rapidly emerging as a potential strategy for tissue repair and regeneration in many fields of medicine. In orthopedics, cartilage repair has played a pioneering role in the translational application of cell therapy, and stem cells have already been used in orthopedic applications in the treatment of avascular bone necrosis, osteochondral defects, pseudoarthrosis, and traumatic cartilage defects [6]. In 2001, Zuk et al. described the human adipose tissue as a source of multipotent mesenchymal stromal cells (MSCs) like those found in the bone marrow [7,8,9]. The regenerative effect is due to the presence of adipose-derived stem cells (ADSCs), cytokines, growth factors, pre-adipocytes, and mature adipocytes, that led to a growing interest in the use of fat graft as a regenerative treatment. In particular, ADSCs show the capacity to differentiate into multiple cell types, including adipocytes, chondrocytes, myocytes, hepatocytes, and endothelial cells, and display the ability to secrete bioactive molecules which stimulate angiogenesis and have antifibrotic, antiapoptotic, and immunomodulatory properties [10,11,12,13].

In this paper, we present our experience in the management of hip and knee OA by means of an intra-articular injection of fat micrograft, describing our approach, which was developed from the belief in the powerful reparative effect of autologous fat graft on damaged tissue, as well as its natural lubricating effect on the joints.

## 2. Materials and Methods

This observational retrospective study was conducted under the Declaration of Helsinki guidelines. As the study was carried out in the senior authors’ private practices, international review board approval was not required. Before any treatment was performed, all patients received detailed information regarding the procedure and provided written consent.

Inclusion criteria were as follows: men and women, aged 20 to 80 years, that referred articular pain of the hips and/or knees, showing initial-stage degenerative OA. OA was radiographically graded according to the Tönnis classification [14] for the hip and the Kellgren–Lawrance (KL) classification [15] for the knee.

The Tönnis grading scale consists of four progressive degrees of degenerative changes to the hip, from grade 0 (absence of arthrosis) to the most severe grade 3 (large cysts, severe narrowing of the joint space, severe femoral head deformity, and avascular necrosis) [16] (Table 1).

The KL classification is typically applied specifically within the context of knee OA and is correlated with an increasing severity of OA, with Grade 0 signifying the absence of OA and Grade 4 signifying severe-stage OA [17] (Table 2).

Patients with an OA of grade 1–2 according to the Tönnis grading scale and grade I–III according to the KL classification were included (as well as one patient with grade IV). All patients had previously undergone conservative treatment, such as anti-inflammatory systemic medicines and/or physical therapy and/or injections (corticosteroids or viscosupplements), without receiving a real benefit on pain.

Exclusion criteria were any patient parameters falling outside of the inclusion criteria parameters and any current oral or parenteral steroid or blood thinner use; patients with deformities, infections, necrosis, and tumors were excluded; and patients with stiffness more then 50% of the articular range of motion were excluded.

Before treatment, each patient had undergone radiologic evaluation with anteroposterior and lateral X-rays of the hip or knee and Magnetic Resonance Imaging (MRI) of the painful hip/knee to evaluate and grade the articular OA that was classified for each patient using the classifications reported above.

Then, we divided the population into a Knee Group (KG) and a Hip Group (HG). For the follow-up, 3 months were consider the minimum time to see results, and then clinical control was performed after 6 and 12 months.

For the analysis of the measurements, it must be taken into account that we treated mild or moderate pathologies conservatively, and so we did not expect differences in mobility as in cases of total replacement for severe stiffness, but small increases in movement. For this reason, we decided to measure the improvement in range of motion as an overall increase greater than 10° (considering abduction + adduction + flexion-extension + rotations). The patient’s orthopedic status was clinically evaluated by the main investigator, prior to injection and postoperatively at 3 months. Joint pain and mobility were clinically assessed by measuring the difference in range of motion (ROM) more than 10°, measuring the stiffness during the visit according to stimulated pain or not, and then evaluating the patient’s ability to rapidly rise from a chair, move rapidly 2 m from the chair, turn, return, and sit without help. Each patient was preoperatively administered a Visual Analogue Scale (VAS) with a 10-point validated pain scale questionnaire [18]. In the postoperative period, the VAS was submitted at 3, 6, and 12 months. Each patient filled in the SF-12 questionnaire at 12 months postoperatively, except patients treated last year. Global patient satisfaction was assessed at the end of the follow-up by asking the patient “are you completely satisfied with the treatment, would you do it again?”. We did not consider repeat postoperative X-rays as necessary. The Microsoft Excel (Excel 16.85) mathematical basis of statistical models was used. The SF-12 questionnaire was filled in by 198 patients (79%).

### 2.1. Technique

#### 2.1.1. The Guided SEFFI Device and Procedure

**Figure 1:**
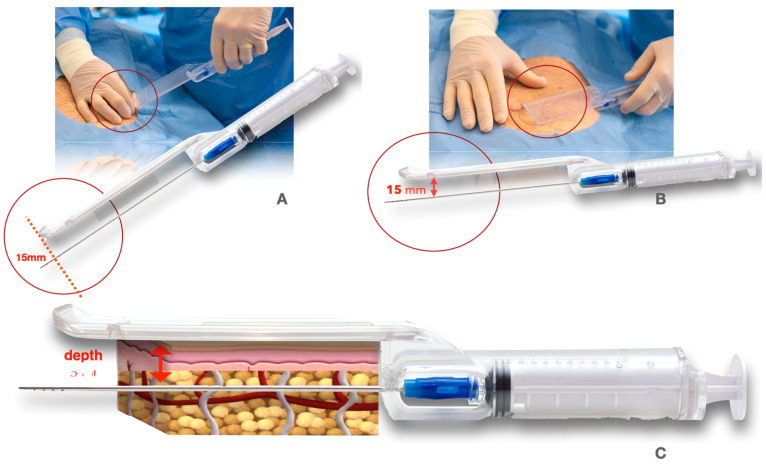
(**A**) Guided cannula introduction (15 mm depth); (**B**) guided tissue harvesting (15 mm); and (**C**) guided tissue harvesting in the SAT (superficial adipose tissue).

Guided SEFFI is the acronym for Superficial Enhanced Fluid Fat Injection and Sefficare™ is produced by SEFFILINE S.r.l., Bologna, Italy; it is a CE-marked class IIA medical device used to perform autologous regenerative treatments in a safe, standardized, easy, and effective way. The device is all-in-one and disposable: it includes all materials needed for the whole procedure and both a harvesting cannula and a guide. The guide aims to standardize the procedure and to guarantee that tunneling is performed in the subcutaneous tissue adjacent to the dermis (15 mm depth); the superficial adipose tissue (SAT) has been proven to hold more mesenchymal and vascular stem cells [19,20] (Figure 1).

**Figure 2:**
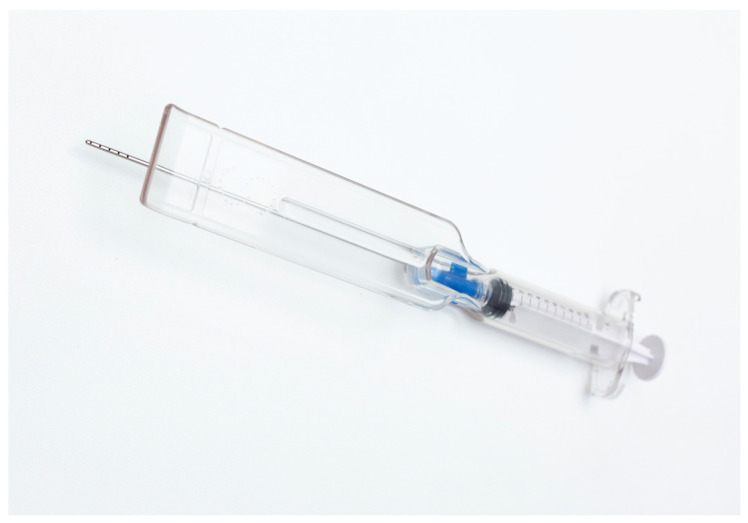
SEFFICARE™ guide and harvesting cannula.

The device aims to collect micro-fragmented adipose tissue naturally containing stromal vascular fraction cells (SVFc) and mesenchymal stem cells ADSCss [21,22,23]. The SEFFI technique is based on the rationale that a highly fluid preparation of adipose tissue clusters can be generated in the harvesting step without any need for further manipulation. Any mechanical tissue manipulation leads to a reduction in stemness and cell viability; a study proved that tissue harvested with this technique (without substantial manipulation) leads to a tissue with higher viability and higher growth rate [24] compared to other techniques involving the mechanical manipulation of the harvested tissue [25]. In fact, the adipose tissue is dissociated in small clusters by harvesting with a cannula with small side-port holes. The Sefficare™ device includes one harvesting cannula (15 cm in length and 2 mm in diameter), provided at its tip with 15 side-port holes of 1 mm that collect the micro-fragmented adipose issue (Figure 2).

All patients were maintained under local anesthesia and monitored. Every procedure was performed under complete aseptic technique and antibiotic prophylaxis for five days after treatment. The surgical site of liposuction was carefully chosen based on the availability of fat and the patients’ wishes, typically from the abdomen, flanks, or lateral thighs. Neither the type of surgical procedure nor the anatomical site of the subcutaneous adipose tissue harvesting significantly affects the total number of viable cells that can be obtained from the SVF.

The anesthetic solution is administered in the selected donor site using the same system (syringe/cannula/guide) in order to standardize the injection in the same plane in which the harvesting procedure will be performed. No tumescent technique is required.

**Figure 3:**
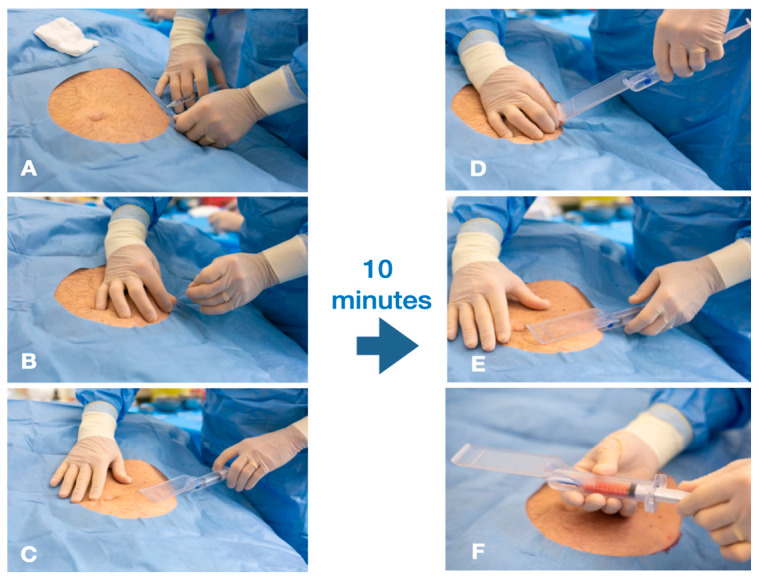
(**A**–**C**) Administration of 30–40 mL anesthetic solution; after 10 min, standardized harvesting procedure; (**D**) guided introduction of the cannula 15 mm under the skin; (**E**) guided harvesting procedure of the superficial adipose tissue (SAT); (**F**) fluid tissue in the VacLock™ syringe.

**Figure 4:**
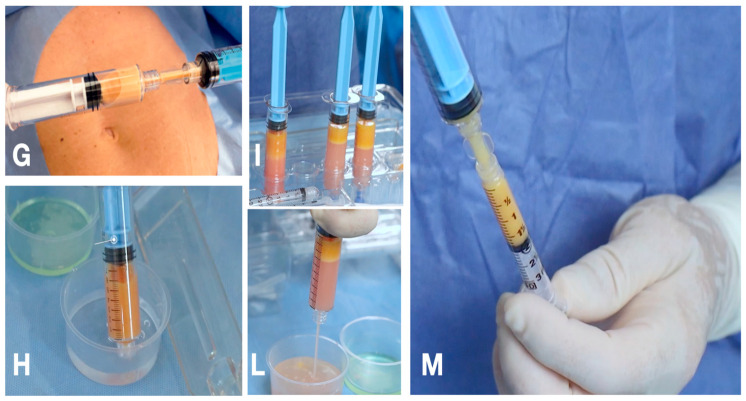
(**G**) Tissue is transferred to decanting syringe; (**H**) the syringe is filled with saline solution; (**I**) the syringes are inserted into the slot for decanting; (**L**) the washing liquid is discharged; (**M**) the tissue is ready to be injected and it is transferred into a smaller syringe (3 mL luer lock).

Saline solution (20 mL) was mixed with lidocaine 2% (10 mL/200 mg), sodium bicarbonate (2 mL), and epinephrine (0.3/mL/mg), and then injected into the selected donor site with a ratio 1:1 of the average amount of harvesting tissue. The superficial adipose tissue (SAT) was manually aspirated using the same system (syringe/cannula/guide), with a target volume of approximately 20 to 30 mL of aspirate (Figure 3 and Figure 4).

The harvesting procedure was stopped when approximately 5–8 mL of tissue was obtained into the harvesting syringe, and then the tissue was transferred into a syringe for decanting; this was then filled with saline solution, capped, and positioned in standing position into the slot for decanting. After an average of one minute, the tissue separated from the washing liquid, the liquid was discharged, and the tissue was ready for injection. The harvesting and washing procedure described above was repeated an average of three times (5–8 mL ×3) in order to obtain the amount of tissue required for the procedure. The tissue was ready to be injected with a 18 G needle; in case a smaller injecting needle is required, it is possible to perform 2–3 passages of the washed tissue from one syringe to another to obtain optimally fluid microcellular clusters, ready to be injected, without any need for further manipulation. Finally, the osteoarthritic joint was injected with autologous intra-articular fat micrograft via needles or cannulas under local anesthesia.

A study proved that tissue harvested with minimal pressure (syringe instead of aspiration device), washed by means of decantation (instead of centrifuge), subjected to minimal manipulation, and minimally exposed to ambient air, results in a higher rate of viable cells [26].

#### 2.1.2. Postoperative Indications and Evaluation

The patient was given a compression bandage for 3 days over the donor site in order to reduce the risk of hematoma, and it was suggested that they do not practice sport for 60 days after treatment.

### 2.2. Statistical Analysis

Count data were summarized as absolute numbers and proportions; continuous data as means and ranges. Data were analyzed using Excel for Mac (Microsoft Corporation Redmond, Washington, DC, USA). No inferential statistical analyses were performed.

## 3. Results

From October 2018 to February 2023, a total of 250 patients underwent treatment with the Sefficare^®^ device. Women were the majority (160 women, 64%; 90 men, 36%). In total, 190 procedures (76%) involved the knees, with 20 patients who were bilaterally treated (every bilateral case was evaluated as if the conditions of the knees were comparable), while 60 procedures, all unilateral, involved the hips (24%). The mean age at treatment was 52.4 years, with an age range of 32 to 80 years.

The Knee Group (KG) included 190 patients, 127 women (67%) and 63 men (33%). Osteoarthritis according to the KL classification had an average score of 1.6; 84 patients (44%) had grade 1 (51 women and 33 men), and 106 (56%) patients had grade 2 (70 women and 36 men).

The Hip Group (HG) included 60 patients, 33 women (55%) and 27 men (45%). Osteoarthritis according to the Tonnis classification had an average score of 1.5; 29 patients (49%) had grade 1 (14 women and 15 men), and 31 patients (51%) had grade 2 (19 women and 12 men).

Postoperatively, every patient was assessed after three, six, and twelve months.

Clinically, the ROM of the treated knees increased an average of 10 degrees after treatment, but the difference in range of motion before and after the treatment was not significant because the OA included in the study was a low-grade one, but results were satisfactory in 139 patients out of 190 (73%) in KG, 96 women and 43 men. The mean age in this positive group was 50.9 years old (range 22–64) and the mean KL classification was 1.4; the VAS at 6 months was an average of 2.0 (range 1–3).

On the other hand, no clinical difference was observed in 51 patients (27%), 31 women and 20 men, in KG; the mean age in this negative group was 59.9 years old (range 45–80) and the mean KL classification was 2; the VAS at 6 months was an average of 2.3 (range 1–4). The same results were deduced in terms of reduction in stiffness (painless movements) and the clinical test of rising from a chair, moving rapidly 2 m from the chair, turning, returning, and sitting without help. The study cohort was not very large and this has several limitations, as there was no control group, but results look promising.

Clinically, the ROM of the treated hip increased an average of 10 degrees after treatment at 3 months after surgery in 45 patients in HG (75%). The increase in movement was measured overall by adding the improvement in abduction, adduction, rotations, and flexion-extension (23 women and 22 men); the mean age in this positive group was 50.9 years old (range 44–61) and the mean Tonnis classification was 1.4; the VAS results at 6 months decreased to a value of 2.1 (range 1–3).

On the other hand, no clinical difference in ROM was observed in 15 patients (25%), (10 women and 5 men) in HG; the mean age in this “negative” group was 57.1 years old (range 51–66) and the mean Tonnis classification was 1.6; however, we observed a reduction in pain expressed in the VAS at 6 months, with an average value of 2.1 (range 1–4).

The same results were deduced in terms of reduction in stiffness (painless movements) and the clinical test of rising from a chair, moving rapidly 2 m from the chair, turning, returning, and sitting without help.

The VAS was submitted at 3, 6, and 12 months, demonstrating a progressive reduction in pain, with the best score obtained at six months postoperatively for KG and at twelve months for HG.

In KG, the mean preoperative VAS was 7.5 (range 7–9), 7.4 for women and 7.6 for men; the 3-month VAS was 5.8 (range 4–7), 5.7 for women and 5.8 for men; after 6 months, the VAS was 2.1 (range 1–4), 2.1 for women and 2.1 for men; and the 12-month VAS was 2.2 (range 1–3), 2.2 for women and 2.2 for men.

In HG, the mean preoperative VAS was 7.35 (range 6–9), 7.4 for women and 7.2 for men; the 3-month VAS was 5.3 (range 3–7), 5.4 for women and 5.3 for men; the 6-month VAS was 2.0 (range 1–3), 2.1 for women and 1.9 for men; and the 12-month VAS was 1.4 (range 1–2), 1.3 for women and 1.4 for men.

The results are presented in Table 3.

The SF-12 questionnaire was filled in by 198 patients (79%), of whom 157 were in KG (83%), where the mean average was 54.36 (range 51–56) for PCS-12 and 56.85 (range 55, 61–58, 74) for MCS-12. The SF-12 was filled in by 41 patients (68%) in HG. The mean average was 54.48 (range 48–56) for PCS-12 and 57,12 (range 54, 61–57, 74) for MCS-12.

The results could be resumed in “Not limited in moderate activities; Not limited in moderate activities; Accomplished as much work as would like (unaffected by physical health); Not limited in any kind of work or other activities in last week (unaffected by physical health); Accomplished as much work as would like (unaffected by emotional problems); Works as carefully as usual (unaffected by emotional problems); No interference of pain with normal work; Had a lot of energy all of the time; Felt downhearted and blue none of the time; In general, health is very good; Felt calm and peaceful most of the time; Physical or emotional health interferes with social activities a little of the time” [27,28,29,30].

We asked patients the question “are you completely satisfied with the treatment, would you do it again?” In total, 212 patients (85%) answered “yes” and were satisfied one year after treatment, with a considerable improvement in pain and quality of life. The remaining 25% continued to follow-up with conservative and surgical therapies in particular: by the five-year follow-up, only 12 patients (4.8%), 11 men and 1 woman, had undergone surgery, in particular, 9 knee replacements and 6 hip replacements; all patients were more than 65 years old and were treated with stem cells in the previous 2 years.

The donor site postoperative course was uneventful other than minimal discomfort, edema, and ecchymosis, and no adverse events, including pain and infection, and no major complications were observed.

The injected site postoperative course was only characterized by swelling and a low-grade pain for 3–7 days; no adverse events or infection were observed in the injected joint. The injected material was well tolerated because it is autologous.

## 4. Discussion

OA is an active disease process with an imbalance between the repair and destruction and degeneration of joints, with poor intrinsic healing power and regeneration due to poor vascularization and the absence of direct access to progenitor cells of the bone marrow. Autologous Regenerative Therapy (ART) is an innovative medical discipline that aims to regenerate injured tissues or to stimulate their repair, using the same natural principles of tissue engineering with an easy, reproducible, and fast procedure. The technique utilizes the patient’s own cells (autologous MSCs) in a single medical procedure. The characteristic of a mesenchymal cell is to differentiate depending on the signals it receives from the surrounding environment and specific growth factors. This ability makes it the ideal element to stimulate the healing of many lesions that involve different tissues. ADSCs are especially found in the stromal vascular fraction (SVF) of the adipose tissue [31], and they are frequently used in the orthopedic field thanks to their intrinsic capacity to regenerate cartilage, tendons, and bones.

In this paper, we presented our cohort of patients, treated for hip and knee OA by means of an intra-articular injection of fat micrograft with Sefficare^®^ devices. (SEFFILINE srl, Bologna, Italy)

We demonstrated that the harvesting of donor adipose tissue, the isolation of the SVF, and its intra-articular injection in knees and hips constitute a relatively simple procedure which can be performed in a session of approximately 60 to 70 min.

**Figure 5:**
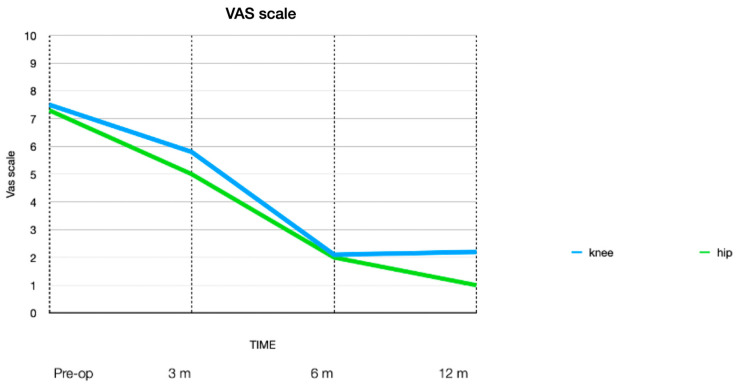
Mean value of VAS.

The study court is not very large and this has several limitations, as there is no a control group, and the technique still appears to be spreading on a large scale; however, we can deduce that the best clinical results were obtained in younger patients with lower OA grade, according to the classifications used. The clinical results, in terms of increase in ROM and reduction in stiffness appear around 3 months, while the reduction in pain measured on the VAS begins at 3 months but reaches its peak after 6 months for the knee and after 6–12 months for the hip (Figure 5). Similarly, the worst clinical results in terms of mobility are observed in older patients and with more severe OA, but even in these cases, a reduction in pain is observed.

In the postoperative period, we observed an improvement regarding globally pain and quality of life of patients, who demonstrated a general level of satisfaction according to other studies [32]. In contrast, neither the type of procedure nor the anatomical site of the subcutaneous adipose tissue harvesting significantly affects the total number of viable cells that can be obtained from the SVF [33].

Clinical improvement in terms of pain reduction and the improvement of joint performance seems most related to a less severe preoperative condition. However, even in the most serious conditions, pain reduction has been observed with good clinical satisfaction [34,35]. Best results in terms of pain reduction have been observed around six months, which means that it takes time for the clinical benefit, while movement and stiffness appear to benefit in the shorter time. Patient’s age evidently correlates with a more severe OA condition; however, there are no particular age limits in which the treatment does not cause a benefit.

It would be advisable to expand the study with a control group and adding more data, for example, the presence of length discrepancy, other systemic pathologies, axis defects, previous trauma, etc., in order to give major and more targeted indications. However, a low complication rate was observed so we can say that treatment with mesenchymal stem cells is recommended as a good option for an improvement in quality of life in patients with mild or moderate OA or, in the most severe cases, for patients who refuse surgical treatment. However, results are variable and not yet predictable [36,37].

The donor site postoperative course was uneventful other than minimal discomfort, edema, and ecchymosis, and no adverse events, including pain and infection, and no major complications were observed.

The injected site postoperative course was only characterized by swelling and a low-grade pain for 3–7 days; no adverse events or infection were observed in the injected joint. The injected material was well tolerated because it is autologous.

The Hip Group patients treated with stem cells appeared to have better results in terms of pain reduction at 6 and 12 months compared to the Knee Group patients treated, but the difference was not significant. We had more female patients, and women always have better clinical results; we also had a lower percentage of patients requiring joint replacement; this may be due to a greater propensity of women for conservative treatments and less postoperative wear (for example, more use of oral supplements, weight control, physiotherapy, etc.). For this and other reasons, other studies with more variables are required; however, the preliminary results obtained appear encouraging.

## 5. Conclusions

With the results we obtained, as previously described in the literature, we confirmed that the use of autologous adipose-derived SVF for the treatment of OA was safe and feasible [38,39,40]. The satisfactory outcome of this minimally invasive procedure indicates that the intra-articular injection of fat micrograft can replace or considerably delay the need for the classical major joint replacement surgery, thanks to its impact on the quality of life of patients and financial cost.

This study presents diverse limitations. Its retrospective nature and the limited number of cases are the main limits. Furthermore, a longer follow-up period may be necessary. In the future, more studies on this treatment are needed.

## 6. Patents

The guide in the SEFFICARE™ device is patented in Italy. 

## Figures and Tables

**Table 1:** Tönnis grading scale of hip osteoarthritis.

Grade	Radiographic Features
**0**	- no signs of osteoarthritis
**1**	- slight narrowing of joint space
	- slight lipping at joint margin
	- slight sclerosis of the femoral head or acetabulum
**2**	- small cysts in the femoral head or acetabulum
	- increasing narrowing of joint space
	- moderate loss of sphericity of the femoral head
**3**	- large cysts
	- severe narrowing of joint space
	- severe deformity of the femoral head
	- avascular necrosis

**Table 2:** Kellgren–Lawrance (KL) classification.

Grade	Radiologic Findings
**0**	no radiological findings of osteoarthritis
**I**	doubtful narrowing of joint space and possible osteophytic lipping
**II**	definite osteophytes and possible narrowing of joint space
**III**	moderate multiple osteophytes, definite narrowing of joint space, small pseudocystic area with sclerotic walls and possible deformity of bone contour
**IV**	large osteophytes, marked narrowing of the joint space, severe sclerosis and definite deformity of bone contour

**Table 3:** Results according to clinical improvement related to OA classification.

	N	Improvement ROM (or Stiffness) Post Operative (n) and VAS				
		>ROM 3-Month	<Stiffness 3-Month	VAS 3-Month	VAS 6-Month	VAS-pre
Knee patients Tot 190						
KL grade 1	84	78	78	5.7	1.9	7.5
KL grade 2	101	61	61	5.9	2.2	7.5
KL grade 3	4	0	0	5.9	2.3	8
KL grade 4	1	0	0	6	2.5	8.5
Hip patients Tot 60						
Tonnis grade 1	29	26	26	5.3	2.0	7.1
Tonnis grade 2	31	19	19	5.3	2.2	7.5

## Data Availability

The data presented in this study are available on request from the corresponding author.

## References

[1] Buckwalter J.A., Martin J.A. (2006). Osteoarthritis. Adv. Drug Deliv. Rev..

[2] Dunlop D.D., Manheim L.M., Yelin E.H., Song J., Chang R.W. (2003). The costs of arthritis. Arthritis Rheum..

[3] Moshref S.S., Jamal Y.S., Al-Hibshi A.M., Kaki A.M. (2019). The Regenerative Effect of Intra-Articular Injection of Autologous Fat Micro-Graft in Treatment of Chronic Knee Osteoarthritis. Tibia Pathology and Fractures.

[4] Mordin M., Parrish W., Masaquel C., Bisson B., Copley-Merriman C. (2021). Intra-articular Hyaluronic Acid for Osteoarthritis of the Knee in the United States: A Systematic Review of Economic Evaluations. Clin. Med. Insights Arthritis Musculoskelet. Disord..

[5] Coleman C.M., Curtin C., Barry F.P., O’Flatharta C., Murphy J.M. (2010). Mesenchymal stem cells and osteoarthritis: Remedy or accomplice?. Hum. Gene Ther..

[6] Peeters C.M.M., Leijs M.J.C., Reijman M., van Osch G.J.V.M., Bos P.K. (2013). Safety of intra-articular cell-therapy with culture-expanded stem cells in humans: A systematic literature review. Osteoarthr. Cartil..

[7] Zuk P.A., Zhu M., Mizuno H., Huang J., Futrell J.W., Katz A.J., Benhaim P., Lorenz H.P., Hedrick M.H. (2001). Multilineage cells from human adipose tissue: Implications for cell-based therapies. Tissue Eng..

[8] Zuk P.A., Zhu M., Ashjian P., De Ugarte D.A., Huang J.I., Mizuno H., Alfonso Z.C., Fraser J.K., Benhaim P., Hedrick M.H. (2002). Human adipose tissue is a source of multipotent stem cells. Mol. Biol. Cell.

[9] Zuk P.A. (2010). The adipose-derived stem cell: Looking back and looking ahead. Mol. Biol. Cell.

[10] Tallone T., Realini C., Bohmler A., Kornfeld C., Vassalli G., Moccetti T., Bardelli S., Soldati G. (2011). Adult human adipose tissue contains several types of multipotent cells. J. Cardiovasc. Transl. Res..

[11] Huang J.I., Beanes S.R., Zhu M., Lorenz H.P., Hedrick M.H., Benhaim P. (2002). Rat extramedullary adipose tissue as a source of osteochondrogenic progenitor cells. Plast. Reconstr. Surg..

[12] Fraser J.K., Schreiber R., Strem B., Zhu M., Alfonso Z., Wulur I., Hedrick M.H. (2006). Plasticity of human adipose stem cells toward endothelial cells and cardiomyocytes. Nat. Clin. Pract. Cardiovasc. Med..

[13] Caplan A.I., Dennis J.E. (2006). Mesenchymal stem cells as trophic mediators. J. Cell Biochem..

[14] Brückl R., Hepp W., Tönnis D. (1971). Differentiation of normal and dysplastic juvenile hip joints by means of the summarized hip factor. Arch Orthop Unfallchir..

[15] Kellgren J.H., Lawrence J.S. (1957). Radiological assessment of osteo-arthrosis. Ann. Rheum. Dis..

[16] Kovalenko B., Bremjit P., Fernando N. (2018). Classifications in Brief: Tönnis Classification of Hip Osteoarthritis. Clin. Orthop. Relat. Res..

[17] Kohn M.D., Sassoon A.A., Fernando N.D. (2016). Classifications in Brief: Kellgren-Lawrence Classification of Osteoarthritis. Clin. Orthop. Relat. Res..

[18] Gould D., Kelly D., Goldstone L., Gammon J. (2001). Examining the validity of pressure ulcer risk assessment scales: Developing and using illustrated patient simulations to collect the data INFORMATION POINT: Visual Analogue Scale. J. Clin. Nurs..

[19] Trivisonno A., Di Rocco G., Cannistra C., Finocchi V., Torres Farr S., Monti M., Toietta G. (2014). Harvest of superficial layers of fat with a microcannula and isolation of adipose tissue-derived stromal and vascular cells. Aesthet.Surg. J..

[20] Di Taranto G., Cicione C., Visconti G., Isgro M.A., Barba M., Di Stasio E., Stigliano E., Bernardinin C., Michetti F., Salgarello M. (2015). Qualitative and quantitative differences of adipose- derived stromal cells from superficial and deep subcutaneous lipoaspirates: A matter of fat. Cytotherapy.

[21] Gennai A., Bernardini F.P. (2017). Superficial enhanced fluid fat injection (SEFFI and MicroSEFFI) in facial rejuvenation. CellR4.

[22] Bernardini F.P., Gennai A., Izzo L., Zambelli A., Repaci E., Baldelli I., Fratertnali-Orcioni G., Hartstein M.E., Santi P.L., Quarto R. (2015). Superficial Enhanced Fluid Fat Injection (SEFFI) to correct volume defects and skin aging of the face and periocular region. Aesthet. Surg. J..

[23] Bernardini F.P., Gennai A. (2016). Fluid fat injection for volume restoration and skin regeneration of the periocular aesthetic unit. JAMA Facial Plast. Surg..

[24] Gennai A., Bovani B., Colli M., Melfa F., Piccolo D., Russo R., Roda B., Zattoni A., Reschiglian P., Zia S. (2021). Comparison of Harvesting and Processing Technique for Adipose Tissue Graft: Evaluation of Cell Viability. Int. J. Regenr. Med..

[25] Senesi L., De Francesco F., Farinelli L., Manzotti S., Gagliardi G., Papalia G.F., Riccio M., Gigante A. (2019). Mechanical and Enzymatic Procedures to Isolate the Stromal Vascular Fraction from Adipose Tissue: Preliminary Results. Front. Cell Dev. Biol..

[26] Cucchiani R., Corrales L. (2016). The Effects of Fat Harvesting and Preparation, Air Exposure, Obesity, and Stem Cell Enrichment on Adipocyte Viability Prior to Graft Transplantation. Aesthetic Surg. J..

[27] Ware J., Kosinski M., Keller S. (1996). A 12-Item Short-Form Health Survey: Construction of scales and preliminary tests of reliability and validity. Med. Care.

[28] Gandek B., Ware J.E., Aaronson N.K., Apolone G., Bjorner J.B., Brazier J.E., Bullinger M., Kaasa S., Leplege A., Prieto K. (1998). Cross-validation of item selection and scoring for the SF-12 Health Survey in nine countries: Results from the IQOLA Project. J. Clin. Epidemiol..

[29] Jenkinson C., Layte R., Jenkinson D., Lawrence K., Peterson S., Paice C., Stradling J. (1997). A shorter form health survey: Can the SF-12 replicate results from the SF-36 in longitudinal studies?. J. Public Health.

[30] Ware J.E., Keller S.D., Kosinski M. (1995). SF-12: How to Score the SF-12 Physical and Mental Health Summary Scales.

[31] Crisan M., Yap S., Casteilla L., Chen C.W., Corselli M., Park T.S., Andriolo G., Sun B., Zheng B., Zhang L. (2008). A perivascular origin for mesenchymal stem cells in multiple human organs. Cell Stem Cell.

[32] Thoene M., Bejer-Olenska E., Wojtkiewicz J. (2023). The Current State of Osteoarthritis Treatment Options Using Stem Cells for Regenerative Therapy: A Review. Int. J. Mol. Sci..

[33] Yu H., Huang Y., Yang L. (2022). Research progress in the use of mesenchymal stem cells and their derived exosomes in the treatment of osteoarthritis. Ageing Res. Rev..

[34] Giorgino R., Albano D., Fusco S., Peretti G.M., Mangiavini L., Messina C. (2023). Knee Osteoarthritis: Epidemiology, Pathogenesis, and Mesenchymal Stem Cells: What Else Is New? An Update. Int. J. Mol. Sci..

[35] Lee W.-S., Kim H.J., Kim K.-I., Kim G.B., Jin W. (2019). Intra-Articular Injection of Autologous Adipose Tissue-Derived Mesenchymal Stem Cells for the Treatment of Knee Osteoarthritis: A Phase IIb, Randomized, Placebo-Controlled Clinical Trial. Stem Cells Transl. Med..

[36] Song Y., Du H., Dai C., Zhang L., Li S., Hunter D.J., Lu L., Bao C. (2018). Human adipose-derived mesenchymal stem cells for osteoarthritis: A pilot study with long-term follow-up and repeated injections. Regen. Med..

[37] Entessari M., Oliveira L.P. (2023). Current evidence on mesenchymal stem cells for hip osteoarthritis: A narrative review. Regen. Med..

[38] Wei P., Bao R. (2022). Intra-Articular Mesenchymal Stem Cell Injection for Knee Osteoarthritis: Mechanisms and Clinical Evidence. Int. J. Mol. Sci..

[39] Davatchi F., Abdollahi B.S., Mohyeddin M., Shahram F., Nikbin B. (2011). Mesenchymal stem cell therapy for knee osteoarthritis. Preliminary report of four patients. Int. J. Rheum. Dis..

[40] Freitag J., Bates D., Wickham J., Shah K., Huguenin L., Tenen A., Paterson K., Boyd R. (2019). Adipose-derived mesenchymal stem cell therapy in the treatment of knee osteoarthritis: A randomized controlled trial. Regen. Med..

